# Report on the Progress of Anatomy and Physiology

**Published:** 1846-03

**Authors:** W. H. Ranking


### RECORD OF MEDICAL SCIENCE.

### *Report on the Progress of Anatomy and Physiology.*

By W. H. Ranking, M.D.

### § VII.—*Generation.*

34. *Analogy of menstruation with the "rut" of Animals, &c.*—Few subjects have had so much attention directed towards them, or have been so fully investigated of late years, as those which have reference to the phenomena of menstruation, the maturation and discharge of ova, and the formation of corpora lutea. All have received considerable elucidation, and the result has been that many highly interesting and important points have been ascertained concerning each, and what was before considered strange and anomalous, is now shown to be all order and harmony. Thus menstruation, which until of late was almost universally regarded as peculiar to the human female, is now proved beyond doubt to be analogous in all essential particulars to the phenomena attending the process of *heat* or *rut* in animals. The investigation of many British and continental physiologists have established the truth of this analogy almost beyond question. Among the most recent labourers on the subject of menstruation is Mr. Girdwood, who, in a capital paper, has summed together most of the facts establishing the above-mentioned analogy, and has, moreover, strengthened these facts by the results of several original experiments. He draws attention to the following conclusions, (the first two of which, however, have been also well established by the researches of Bischoff, Raciborski, and other recent inquirers.) 1st. The catamenia appear in lower animals as well as in the human female, and whilst the discharge is characterized in them as in her, by a periodicity peculiar to each separate genus, it is (at least in the higher orders of mammals) equally sanguineous in them as it is in woman. From observations on the dog, rabbit, cow, and mare, Mr. Girdwood proves that the occurrence of a periodic discharge in them attends the phenomena of heat, and the discharge microscopically and chemically bears a more or less close resemblance to that of the human female, and he observes that in passing up through the series of animated beings, it would appear that the nearer we approach man, the more the catamenial discharge approximates in character that of our own species. It appears in all to consist of a profuse periodic flow of the usual mucous secretion of the organs of generation, with, in the higher genera of animals, the super-addition, generally, of more or less blood diffused in the secretion, and from this its diffusion deprived of its usual amount of coagulation. 2d. In woman, and in the females of all animals in which the periodic discharge is apparent, the discharge is indicative of the maturation of an ovum, and of its being on the point of elimination from the ovary ; and the capability for impregnation is, during menstruation, at its acme. This corresponds with the observations of Bischoff, Pouchet, Raciborski, &c., and is now a generally adopted principle ; for although, on account of the minute size of the human ovum and the rare opportunities afforded of examining the bodies of women who have died during the menstrual period, it is difficult to prove the question beyond doubt by the discovery of ova, yet sufficient observations have been made to render it pretty certain that the above observation is correct, so far as the human female is concerned. With regard to animals, numerous observations and experiments, especially those by Bischoff and Raciborski have rendered it quite certain that at every period of heat one or more ova are matured and discharged from the ovary, and this moreover quite independent of sexual intercourse. The statements of these eminent physiologists are founded on the surest of all evidence, the actual detection of ova in the fallopian tubes of animals in whom sexual intercourse had not taken place ; thus Bischoff found in a lamb, killed a few hours after becoming in rut for the first time, (and in whom coition had not been effected,) a ruptured Graafian vesicle in the right ovary, and an ovum in the corresponding fallopian tube. Again, in a bitch two days after becoming in heat, and apparently inclined to admit the male, (which it was prevented from doing,) Bischoff extirpated the left ovary and fallopian tube, and closed the wound by suture ; no Graafian vesicle in this ovary had burst, but four were extremely turgid. Five days afterwards the bitch was killed ; four large corpora lutea were found in the right ovary, and four ova in the corresponding fallopian tube.

From this it appears quite certain, as Bischoff concludes, that during the rut of animals, ova become detached from the ovary and enter the fallopian tube where they perish, unless sexual intercourse coincidly occurs, in which case the ova in all probability become impregnated. The same may be considered to hold good in the human female. Premature appearance of the catamenia seems to depend, according to Raciborski, on the precocious developement of the ova; a circumstance in favour of this opinion is the invariable coincidence of developement of the mammæ and external genital organs with the occurrence of the hemorrhage, in cases of premature menstruation. There are still wanting observations to show what is the exact purpose served by the periodical discharge of a sanguineous fluid attending the maturation and discharge of ova; numerous ingenious suggestions have been made, but the right one does not seem yet to have been hit upon. There seems no doubt but that all the phenomena of menstruation are primarily dependent on the ovaries, which act sympathetically on the uterus, as well as on other organs. A main argument which has been advanced against menstruation in the human female being a process identical with the rut of animals, is the disinclination to sexual intercourse evinced by woman during the menstrual period, whilst it is only during the period of rut that animals will admit the male; but this Bischoff (as also Mr. Girdwood) considers to be the result of habit and the natural delicacy of the sex, rather than of actual disrelish; and it is most probable that in woman as well as in the females of animals, the desire for sexual intercourse is greatest at the menstrual period or heat, especially towards the decline of the discharge, at which latter period, from observations on animals, it is proved that the ova are usually discharged; bitches are generally observed to be languid and to refuse the male during the first few days of heat, but after this they become lively and readily admit of being lined; so, analogous to this is the ailment of the human female during the early part of each menstrual period, and previous to the discharge becoming fully established. 3. A third conclusion arrived at by Mr. Girdwood, in the above-mentioned paper, is, that the periodicity in the maturation of ova and of the attendant phenomena of menstruation is applied to most of the leading phenomena of nature which are dependent upon heat, and that like these it is influenced by seasons. This is ingenious, but wants more extended observations for its establishment.

35. *Discharge of matured ova, &c.* Besides proving that at each period of heat in animals ova are regularly discharged, whether sexual intercourse coincidently takes place or not, Bischoff has added some curious observations, showing that the bursting of the Graafian follicles, and consequent discharge of ova, is entirely independent of sexual orgasm, or of the influence of spermatozoa; thus: (*a*) Coitus may take place, and examination of the ovaries, six, eighteen, or twenty hours afterwards, prove that no ovum has escaped, although spermatozoa may have reached the ovaries. (*b*) Examination immediately after coitus may detect ova which have advanced two or more inches along the fallopian tube, to have done which they must have started some considerable time before the act of coition. Bischoff considers it to be immaterial at what part of the fallopian tube the spermatozoa and ovum come in contact; so that they meet before the ovum has reached the uterus, impregnation is sure to take place. He states that he has clearly traced live spermatozoa up to the ovaries; this statement, and the inference he deduces from it, that fecundation of the ovum may take place at any part of the fallopian tube, is called in question by M. Pouchet, who has recently offered a table showing hourly the progress and condition of the spermatic fluid in the generative apparatus of the mammiferous female. His experiments were chiefly made on rabbits. From the 16th to the 25th hour after copulation, live spermatozoa were constantly found in the vagina and uterine horns. Even to the 21st or 23d hour these animalcules [?] continued very active, but shortly after that, they lost their vivacity, and died towards the 25th hour; so that nothing but broken-up tailless spermatozoa could be seen. He was never able to see live spermatozoa reach more than a very short distance up the uterine extremity of the fallopian tube; and he considers the observations of Bischoff and Wagner, who had found live spermatozoa on the ovaries, to be erroneous. According to his own view, it is in the uterus alone, or probably also at the mouth of the fallopian tube, that impregnation of the ovum can be effected. [To this it can only be said, that the positive evidence of Bischoff ought to receive more consideration that the negative statement of Pouchet, and that if the latter be correct in his views, it will be difficult to account for the mode in which extra-uterine or ovarian impregnation takes place, whilst the observations of Bischoff explain it at once.]

36. *Corpora lutea.* The great importance, in a medico-legal point of view, of determining the true nature of the "corpus luteum," and of ascertaining how far its existence could be safely relied on as proof that sexual intercourse and consequent conception had taken place, has of late years led to numerous investigations on the subject. Although the descriptions of corpora lutea given by most observers differ somewhat from each other, yet, on the whole, they so far correspond that, generally speaking, there can be no great difficulty in pronouncing whether or not a given substance found in an ovary be a true corpus luteum; such a one, at least, as is formed when a matured and discharged ovum has been impregnated. From Dr. Ritchie's observations, it would seem that one cause of the discrepant accounts given of the corpora lutea, may be found in the varying characters which these structures occasionally present; thus concerning the seat of the yellow deposit, about which there has been so much dispute, Dr. Ritchie states that it may be situated either on the exterior of the ruptured Graafian follicle, between its layers, or within its interior; he considers the yellow mass to be an hypertrophy of the granular layer lining the internal membrane of the follicle. Besides varying in seat, Dr. Ritchie states that the corpora lutea also vary a considerable degree in aspect and character, some being of a white colour (*corpora albida*,) and either of a soft fatty aspect, or dense and shining; others (*corpora cephaloidea*) are yellowish, and of a brain-like character; and another class (*corpora rubra*) which are at first similar to the last, although they are plumper, more vascular, better developed, and the granular matter of which they are composed subsequently becomes of a decided red colour. There has always been considerable difficulty in deciding the question, whether or not the presence of a so-called true corpus luteum could be relied on as a proof of conception. Many have considered it to be an infallible test, and have based their evidence upon it, in medico-legal inquiries; but some late observations of Bischoff and Raciborski seem to prove, that not merely as a result of conception, but at each menstrual period, the discharge of an ovum was followed by the formation of a corpus luteum; in this conclusion, however, there is no doubt they were mistaken; and by the following recent observations, Raciborski admits that they were so, although Bischoff still maintains that what he has observed to take place in animals, namely, the formation of a true corpus luteum after each period of heat, takes place in the human female also; this offers a good instance of the danger of drawing conclusions from analogy alone, for although it appears certain that a corpus luteum is formed at each period of heat in animals, which is undistinguishable from that formed after conception, yet any one who has had frequent opportunities of examining human ovaries will be aware how seldom true corpora lutea are found in them, and that they only exist in cases of pregnancy, or where delivery has but recently taken place; whereas, if, as Bischoff supposes, a corpus luteum is fouud at each menstrual period, one ought to find several in almost all the ovaries of those who die during the age in which the habit of menstruation continues. The clots of blood in various stages of discolouration (according to their age,) so commonly found in ovaries, although they indicate a ruptured Graafian vesicle and the discharge of a matured ovum, yet they bear no resemblance or analogy to the yellow mass observed when the discharged ovum has become impregnated.

The following conclusions of M. Raciborski are especially important since they correct his previous statement that a corpus luteum is formed at each menstrual period in the human ovary, and that its existence cannot be regarded as a proof of conception. His present views confirm the observations of Deschamps.

1. The corpus luteum is the result of an hypertrophy of the granular layer lining the internal or proper membrance of the Graafian follicle; the only difference between the corpus luteum and this granular layer is that the granulations of the former are larger and more numerous, and contain many more yellow oil globules. 2. The transformation of the granular layer into a corpus luteum commences as soon as the ovum is matured and the follicle ready to discharge it. 3. As soon as the Graafian follicle is ruptured, the process of transformation becomes more active; but in the human female the degree of activity varies greatly, according as the expulsion of the ovum is spontaneous, as during a menstrual period, or coincides with sexual intercourse, and is followed by conception. In animals this difference does not exist; whether expulsion of the ovum has been followed by coition or not, a true corpus luteum forms. In the human female, however, if expulsion of the ovum has not been followed by conception (as occurs at each menstrual period) the granulations certainly increase, but their activity of growth stops short when they have formed a thin, yellowish membrane, which lines the inner tunic of the vesicle, and is found to contain within it a clot of blood, more or less altered, according to the period at which it is examined. If, on the contrary, conception coincides with expulsion of an ovum, the granulations rapidly increase in size and number, and shortly form a yellow mass, quite filling the cavity of the follicle, or leaving at the most a white fibrinous-looking streak in the centre, indicating the existence of a former cavity. 4. In all cases this corpus luteum remains of its full size to the end of pregnancy; but after delivery it rapidly disappears, so that at the end of three months nothing but a small and almost colourless spot will remain. 5. It results from the above that by simple inspection, one can readily distinguish cases of simple spontaneous expulsion of ova from those in which the expulsion has been followed by conception.

37. *Structure of the uterus.* The following observations on the arrangement of the muscular fibres of the uterus, by M. Jobert de Lamballe, are extracted from the American Journal of Medical Sciences.

The fibres of the single muscle which forms the uterus are arranged in layers, and present the following direction: the longitudinal superficial fibres, which may be called median from their position, are seldom seen on the anterior surface, but are constantly met with on the posterior, where they constitute two superincumbent layers. 1. Posteriorly they begin at the fundus of the uterus, and end at the uterine extremity of the vagina, to which they become attached, with the exception of some few that terminate on the neck of the uterus; they adhere by one surface to the peritoneum, by the other to the oblique fibres. 2. The anterior superficial fibres do not pass along the entire extent of the uterine parietes, but cross each other before they arrive at the round ligament of the opposite side. Some contribute to form this ligament, whereas others pass behind, and terminate on the lateral regions where they cross those of the posterior region. 3. There are other superficial fibres, only evident during pregnancy, which are destined to the fallopian tubes, and to the ovarian ligaments. Some originate at the fundus of the uterus, unite to those which contribute to form the fallopian tubes, and pass on to the anterior part of the ovarian ligament; others, more numerous, originate from the posterior surface of the fundus, and pass on to the same ligament. Lastly, a few transverse fibres from the posterior surface, form its inferior part. The numerous fibres which pass on to the fallopian tubes originate at the fundus of the uterus, and form a thick fasciculus, which divides into two secondary fasciculis, destined one to the ovarian ligament, the other, more voluminous, to the fallopian tube. Some fibres separate from the common fasciculus, and lose themselves in the cellular tissue which separates the fallopian tubes from the round ligament. The deep fibres are very visible when the uterus has undergone rather lengthened boiling. They all evidently present a semi-circular direction, are rather oblique, and only differ from those above described by their small size, and by their belonging exclusively to the body and to the neck of the uterus. They cross each other on the median line anteriorly and posteriorly, as also on the sides, so as to produce a kind of network. Their thickness varies as they approximate the internal surface of the uterus, where they appear to describe circles exterior to the internal membrane. There are annular fibres along the fallopian tubes, which do not entirely encircle it, and are deep seated. Lastly, the blood-vessels are encircled by fibres similar to the deep muscular layer which surrounds the intestinal canal. The uterine neck is formed by fibres which constitute semicircles, and decussate without mingling. The muscular fibres of the vagina are posteriorly continuous with the longitudinal fibres of the uterus, but anteriorly they terminate abruptly, at the junction of the vagina with the uterus.

In order to empty the uterus of its contents, the longitudinal fibres tend to diminish the length of the uterus, whilst the semicircular ones diminish its cavity in every other direction.

38. *Structure of the human placenta.* In giving an abstract of the following observations on the structure of the placenta by Mr. Goodsir, it will render the subject more intelligible to divide it into three heads, as adopted in the original memoir :—

1st. Each placental tuft consists of a trunk, of primary branches, and of secondary branches or villi. Each villus is made up of the following parts : (*a*) An *external* fine transparent membrane. This membrane is common to the whole tuft, passing from one villus to another, and closely covering the free surface of each. (*b*) A layer of flattened nucleated cells beneath this membrane, (*external cells* of the villus,) here and there these cells are grouped together into heaps, in the centre of which is a germinal spot, which is engaged in the constant formation of new cells.* It seems probable that the internal aspect of this layer or cells is lined by a fine membrane, as in the case of the intestinal epithelium. (*c*) Beneath these structures, and immediately surrounding the blood-vessels within the villus, is another still finer and more transparent, but firm and strong membrane, (*internal membrane* of the villus.) This is readily separable from the layer of cells described : the space between them is probably occupied by a peculiar fluid. (*d.*) Within this membrane are the blood-vessels of the villus, consisting of one or sometimes two vessels, which form a simple or contorted loop occupying the cavity of the villus ; they are derived from the umbilical arteries and veins ; they differ from capillaries in their large size, and from arteries and veins

*Vide nutritive centres.

in preserving the same mean diameter throughout: one such vessel occasionally passes from one to two or more villi, forming a loop in each, before it becomes continuous with a vein. (*e*) Between these vessels and the internal membrane are some other cells, nucleated and highly transparent, called the *internal cells* of the villus.

2d. (*a*) The substance of each tuft of the chorion is made up of nucleated cells of various sizes, containing a granular fluid. (*b*) The surface of the tuft is covered by a fine membrane, which consists of flattened cells united by their edges. (*c*) The free extremity of each villus of the tuft is bulbous, and consists of transparent cells arranged round a central germinal spot. These groups of cells are the active agents by which the villi grow. (*d*) As gestation advances, and the allantois becomes applied to the internal surface of the chorion, blood-vessels become developed within the villi, which then communicate with the umbilical vessels. (*e*) Thus, then, the villi of the chorion form the internal (or fœtal) portion of the placental villi, previously described,—the loops of vessels, internal cells, and internal membrane of which have their origin in the villi of the chorion.

3d. (*a*) When impregnation has taken place, the mucous membrane of the uterus becomes greatly developed; the epithelial or cellular secretion of its follicles becomes augmented, and the vascular network occupying the outer follicular spaces becomes increased in size and extent. By this means a new layer or membrane is produced, the *membrana decidua*, which consists of two portions, the thickened vascular mucous membrane and the non-vascular cellular substance secreted by the follicles. The former constitutes at a later period the *decidua vera*, the latter the *decidua reflexa.* (*b*) As the (impregnated) ovum reaches the uterus, the developed mucous membrane or decidua begins to secrete, the os uteri becomes plugged up with a portion of the secretion, and the cavity of the uterus is filled with fluid—around the ovum this secretion consists of spherical nucleated cells, which possess the power of undergoing further development after being detached from the germinal spots or membrane of the secreting organ. These cells around the chorion of the ovum come to constitute the *decidua reflexa.* (*c*) Thus the tufts of the chorion are imbedded in a mass of nucleated cells, which cells are constantly being secreted from the follicles of the uterus, and which in all probability contain within them as they become fully developed, the nutritive materials, which the absorbing cells of the villi of the chorion are constantly taking up for the nourishment of the ovum. This cellular secretion seems thus to be to the ovum of the mammal what the albuminous fluid is to the ova of oviparous animals. (*d*) As the ovum increases in size, the amount of nutriment absorbed by the cells alone, is not sufficient for its wants; the allantois becomes applied to the inner surface of the chorion, and blood-vessels become developed within the tufts and villi. The vessels of the decidua vera at the same time enlarge and assume the appearance of sinuses encroaching on the space formerly occupied by the cellular substance of the decidua reflexa, in the midst of which the villi of the chorion are imbedded. Thus the lining membrane of the vascular system of the mother becomes the *external membrane* surrounding the villi of the placenta. It lines the whole placental cavity, passing from tuft to tuft, and villus to villus, forming in this way threads and bands of venous membrane, which are tubular and filled with cells. These cells are continuous in the one direction with the *external cells* of the placental villi, and in the other with the gelatinous cellular substance constituting the *parietal portion* of the placental decidua, which is in connection with the wall of the uterus. The *central portion* of the placental decidua consists of the external cells and external membrane of the placental villi.

It appears from the above:—

1st. That the placental tufts and villi are made up on the one hand by the tufts and villi of the chorion, comprising umbilical vessels, internal membrane, and internal cells; and on the other hand by the lining membrane of the maternal vascular system, with a layer of cells beneath it, comprising the external membrane and external cells—the first portion is peculiar to the fœtus, the latter to the mother.

2d, These external cells are the remains of the decidua reflexa; they are still continuous with the cellular substance of the parietal placenta, by means of the cells filling the tubular threads of venous membrane.

3d. The function of the external cells is to secrete from the maternal blood (from which they are separated only by the external membrane) the materials of nutrition destined for the fœtus; this function is analogous to the digestive one performed by the intestinal mucous membrane in extra-uterine life.

4th. The function of the internal cells or those belonging to the fœtus is to absorb through the internal membrane the materials secreted from the maternal blood by the external cells. This matter is then taken up by the umbilical vessels and carried away for the nourishment of the fœtus. These internal cells perform a function analogous to that affected in extra-uterine life by the absorbing chyle-cells of the intestinal villi.

5th. Hence the placenta discharges not only the functions of a lung, but also of an intestinal canal to the fœtus.

39 *Respiration of the embryo.* MM. Baudrimont and Martin St. Ange having continued their researches on the absorption of oxygen by ova during their embryonic development, found in all their experiments on the eggs of hens, turkeys, adders, lizards, and many species of batrachians, that there occurred in each a true respiratory process, consisting in the absorption of oxygen, and the exhalation of carbonic acid, nitrogen, and watery vapour.

[It would appear that this process is essential to the development of the embryo. The exhalation of carbonic acid and watery vapour is, no doubt, the result of combustion of carbon and hydrogen by means of the absorbed oxygen; the effect of which will be the generation of a certain amount of heat, independent of that afforded by the mother in the case of birds, and will in part account for the elevation of temperature above that of the surrounding air, observed in some reptiles during incubation.]

40. *Meconic membrane.* Dr. Ridge, in a recent work, describes the existence, during fœtal life, of a membranous sac lining the mucous membrane of the whole alimentary canal, and serving for the "envelopment and security of the meconium." He gives it the name of "membrana meconii." What purpose could be served by such a membrane, which is not adequately performed by the intestinal mucus, it is difficult to conceive. Most probably a compact layer of such mucus investing a portion of meconium has deceived Dr. Ridge into the belief of having discovered a new tissue. Not one alone, however, for he mentions a "rete vasculare," which he describes as a net-work of the most delicate blood-vessels, situated between the membrana meconii and the true mucous membrane. The existence of this vascular network, and consequently of the office attributed to it, of maintaining the nutrition of the membrana meconii, is, we must confess, as improbable as the existence of the membrane itself.

§VIII.—*Miscellaneous Subjects.*

41. *Effects of extirpation of the spleen and thyroid gland.* From Mr. Bardeleben's experiments it results, that the animals which survive the extirpation of the spleen appear speedily to recover their health, and present no difference from those which have not undergone this operation. He never remarked that they were more voracious than other animals, though it has been said that voracity is generally produced. In no case was the organ regenerated, though Meyer of Bonn has observed this. After removal of the thyroid the health was not sensibly affected. In one rabbit, the venereal appetite was considerably augmented; a circumstance worthy of remark, seeing that it has often been asserted, that removal of that gland abolished the venereal appetite. The animal deprived of both spleen and thyroid gland presented no change in any function. This fact is in opposition with the opinion of Tiedemann, that the lymphatic glands and thyroid body performed the functions of the spleen when that organ was extirpated. Finally, some physiologists have advanced the opinion that extirpation of the spleen produced augmentation of the venereal appetite, but abolition of the procreative faculty. M. Bardeleben satisfied himself of the incorrectness of this opinion, by breeding with dogs which had both spleen and thyroid extirpated.

Professor Meyer of Bonn, has offered further proof that after extirpation of the spleen the small lymphatic glands in connexion with the splenic artery become enlarged, coalesce, and, in no long time, form masses of considerable size, which doubtless perform to a certain extent the functions of the extirpated organ. In ducks and hens ten months sufficed for the production of a glandular mass equal in size to the original spleen. This speedy formation of a new organ discharging the functions of a spleen, will account in part for the trifling subsequent derangement resulting from its extirpation.

42. *Parasitic animalcules in the sebaceous follicles.* M. Gruby gives an account of these animacules, the existence of which was discovered by the late M. Simon.

In man this parasitic insect is met with most commonly in the sebaceous glands of the skin of the nose. It usually occupies the excretory duct of the gland, and when there is a hair, the insect surrounds it. Its head is directed towards the base of the gland, its tail towards the surface of the skin, its feet are applied to the internal wall of the excretory duct. The duct is usually dilated at the part where the animal is lodged. In young persons there are never more than two or four parasites in each gland, but in persons of twenty-five and upwards, there are from four to eight. In still older individuals there may be from ten to twenty, in which case almost all the sebaceous glands are affected. They are common to most persons whether in good health or in disease.

It is only in large numbers in the glands that any irritation results, and then they produce that red, elevated, and tender spot at the orifice of the duct so commonly seen about the nose. These parasites exist abundantly in dogs and produce a very formidable and contagious disease. M. Gruby gives an anatomical and zoological description of these animalcules.

43. *Influence of hot air on animal life.* The results of some late experiments by Magendie, prove that no animal can endure the temperature of its body being increased more than 9° F. beyond its natural state. Thus two rabbits, the natural temperature of whose body is 102° F., were severally placed in stoves, the one heated to a temperature of 140°, the other to that of 212°. After a short time the temperature of both rabbits rose to 111° F., that which was in the hottest stove being the first to attain that temperature. He repeated the same experiment many times on different animals, but in no case did the temperature of the animal increase more than nine degrees. The same occurred with birds. Having attained this increase of temperature, the animals and birds in every case soon died; their arterial blood was found dark, like venous blood, did not redden on exposure to air, and had lost its property of coagulating.

The increase of temperature seemed to be attained chiefly through the medium of the skin, for when the head of an aminal was confined in the heated stove, so that it breathed the hot air, the elevation of temperature was much less in an equal period of time than when the body was exposed to the heated air, and the head was out of the stove. Thus a dog, whose body was within the stove, but the head out, lived only twenty-two minutes; but another, whose head was within the heated stove, and the body out, lived forty minutes.

An animal placed in a dry heated stove loses weight, but the amount lost is proportioned to the length of time the animal remains within the stove, not to the degree of heat, and the loss is no greater at the temperature of 212° than at one of 140° in an equal space of time. In stoves heated with moist air, on the other hand, Magendie found that instead of losing they often gained weight. Death occurred much sooner in these stoves than in the ones heated with dry air.

[The result of these experiments are especially interesting when compared with the observations made by M. Constantine James regarding the effects on the body of the hot moist air of the baths or stoves of Nero, at Pozzuoli. The greatest amount of heat which M. James could endure in the hot moist air of the passage leading to the springs was 122° F.: when he had arrived at that part of the passage where the atmosphere had attained this temperature, he was utterly exhausted, and had nearly lost his consciousness. He was almost suffocated at a temperature of 112° F., whereas, in the caves of Lastaccio, in which the heated air is dry, he was enabled to bear without much discomfort a temperature of 176° F.]

44. *Anatomy and use of the thymus gland.* On this subject we are fortunate in being able to refer to some recent researches by Mr. Simon, the value and importance of which has attained for them the high honour of gaining the first Astley Cooper prize. In the endeavour to place before our readers a brief abstract of this work, we shall omit the author's very concise and accurate history of the labours of former writers, and proceed at once to the discussion of the original portions of his labours.

The first appearance presented by the gland, as observed in the fœtal calf, is that of a simple tube lying along the carotid vessels, and exhibiting faint traces of commencing areolar tissue. The contents of the tube at this time are granular, but do not contain any distinctly formed corpuscles. Mr. Simon suspected that this tube was not the primary condition of the organ, but that it might exist at an earlier period in the more simple form of a string of primordial cells; he has not, however, been able to verify the suspicion. He refutes the opinion of Arnold, (*Lehrbuch der Physiologie*, tom. ii. p. 265,) that the thymus is a development of the respiratory mucous membrane, as well as that of Bischoff, that it is in some way connected with the thyroid gland. The development of the gland proceeds in the same manner as that which has been observed as the primordial tube of the true glands, that is to say, by the addition of diverticula, which spring from the sides of the tube. These diverticula, when they have arrived at three fourths of a sphere, themselves give rise to secondary bulgings, which again reproduce others, until at length by the repeated occurrence of the same process conjoined with a continued interstitial molecular increase, the organ attains the bulk and complexity of the structure exhibited by it in the mature state of the fœtus.

The researches of Mr. Simon confirm in the main the dissections of Sir A. Cooper, with respect to the existence of a central cavity; he thinks, however, that it has hitherto been supposed to be larger than it really is, They likewise accord with those of Haugsted, in reference to the period at which the thymus attains its greatest size, this being, not as is commonly supposed during intra-uterine life, but at a certain period after birth. This exact time it is not easy to ascertain, as it is probable that it varies in different instances; it has, however, been laid down as a law by the author, that its bulk is inversely as the amount of mortality and consequent exhaustion of tissue, and its duration, therefore, dependent upon the period at which muscular activity becomes established. In reference to this point the author has arrived at the following results:—1st. During the period next succeeding birth, the activity of the thymus is remarkable; it increases considerably in size, becomes turgid with secretion, and its specific gravity is lowered by the greater fluidity of its contents. This first growth is far out of ratio to the general increase of the body. 2d. For several months it continues to increase at a diminished rate, and merely in proportion to the general growth of the body; its further enlargement ceases about two years after birth. 3d. From this time, during a *very variable* number of years, it remains stationary, and, supposing the individual to be adequately nourished, gradually assumes the structure of fat. 4th. The duration of its decay, and the epoch of its entire vanishing are still more uncertain; about puberty, it seems in most cases, to suffer its chief loss of substance, and to be reduced to a vestigiary form.

The first appearance of this organ before birth is supposed by anatomists to be as early as the fifth week after conception, but in the tenth week of pregnancy it is sufficiently perceptible to the naked eye. It, at this time, exhibits a distinct tubulo-vesicular structure. The third chapter of the work contains a description of the mature gland. Its mode of formation has been already alluded to; it remains only to mention the intervesicular structure and the contained fluid. The intervesicular tissue is a prolongation of the wall of the original tube, and consists of an indescribably fine membrane, over which a close capillary network is spread for the purpose of supplying materials for secretion. This secretion consists of a fluid in which, as was discovered by Hewson, microscopic corpuscles were seen to float. These corpuscles are circular discs of nearly the same size as the coloured particles of the blood. Their average diameter is $\frac{1}{3830}$ of an inch. They are marked by minute dots which are supposed to be molecules of fat in combinatian with fibrine or solid albumen.

The author gives three separate chemical analyses of the thymus fluid, all of which concur in demonstrating the error of the opinion that it was essentially a highly carbonaceous product. It is proved by them on the contrary that the fluid contains no more carbon than enters into the composition of muscle and blood.

The nerves of the thymus are derived from the inferior and middle cervical ganglions and from the cardiac branch of the pneumogastric nerve.

In the comparative anatomy of the gland, the author's researches have been very extensive, but our space will not allow of a repetition of the different tribes of animals in which he has caried on his investigations, we shall content ourselves with giving the following summary of the results to which they lead. 1st. The *presence of the gland* is co-extensive with *pulmonury* respiration. 2d. Its shape and position are variable and unimportant. 3d. Its *size* and *duration* are, generally speaking, in proportion to the habitual or periodical activity of the animal 4th. Where it remains as a persistent organ (as in the hybernating tribes) it is one of the general reservoirs for the accumulation of nutritive material.

In further prosecuting the developmental anatomy of his subject, the author next passes in review the maphological history of the true glandular system, with which he contrasts that of the thymus and its analogues, the thyroid, supraenal glands, and the spleen. The principal difference between the two orders of organs appears to consist in the ultimate arrangement of their secreting cells, that of the true glands being distinctly cellular, that of the glands without ducts, consisting of the cytoblast alone, the involving cell-structure being only of exceptional formation. It is a curious fact, however, that in those animals in which the thymus becomes a permanent organ, the nucleus instead of being simply surrounded by aggregate molecules, as in the temporary state of the organ, is converted into a perfect cell. These different points are rendered plainly intelligible by the plates with which Mr. Simon's work is liberally illustrated.

We now pass on to the most remarkable part of the work, the physiology of the gland. It is thus stated by Mr. Simon:—

"It secretes into a closed cavity certain particular elements of nutrition, which are deposited differently under different circumstances, viz.:—1st. In most animals it occurs only temporarily, the secreted matter then presents itself under a *fluid form*, and closely resembles the liquor sanguinis in ultimate chemical composition. 2d. In some animals, after discharging this temporary function, it assumes one of greater permanency, the sequestration of material in the form of solid fat. In both cases, however, though peculiar, the function is especially the same, and consists in the laying by of nutrient material. How this is used up, Mr. Simon next proceeds to show. Here, however, we are called to notice a certain circumstance which is co-existent with both the temporary and the permanent function of the gland, viz., that in both, *waste of tissue* is at a *minimum*. In the younger animal, muscular activity, which mainly contributes to this waste, has not commenced; in the hybernating animal it is suspended. Now the waste of tissue being at a minimum, the pabulum for the support of the respiratory process, must be supplied from some other source."

This source Mr. Simon declares to be the nutritive matter laid up in the central cavity of the thymus as in a reservoir, and he therefore assumes the office of that gland to be that of sequestrating nutritive matter, whereby it becomes "a sinking fund of nourishment in the service of respiration."

We must apologise for this imperfect analysis of this really valuable publication; our excuse must be that it only came to hand while the preceding pages were in the press. We were anxious that no time should be lost in making our readers acquainted with its new and important contents, otherwise we should have postponed its consideration for our next volume.

Upon the subject of the use of the thymus gland, we have next to mention a recent essay by Dr. Picci, a review of which appears in the *Medico-Chirurgical Review*, of January, 1845; as will be seen, he recurs to a theory propounded nearly two thousand years ago. After glancing at the theories of his predecessors, Dr. Picci suggests that the use of the thymus is chiefly of a mechanical nature, viz., to occupy a certain space within the thoracic cavity, while the lungs remain unexpanded in the fœtus; and thus to prevent the ribs and sternum from falling in too much upon these vital organs. The size of the thymus is inversely as the volume of the lungs; and when the latter become dilated after birth by the admission of air into their cells, the former immediately begins to shrink and becomes atrophied. In truth, it is only in the adult that the thoracic parietes are moulded completely upon the lungs; for in infancy and youth it is rather the thymus that is, in their place, moulded upon the thorax. The situation of this gland, the very nature of its tissue, and the greater expansion and development of its inferior half are adduced as arguments in favour of this opinion, besides the well known circumstance that, in those new-born children in whom the thorax is very largely developed, the thymus continues to increase gradually even to the end of the second year, it deserves notice that all those animals, in which the lungs are similar to those in the human subject, are provided with this gland; whereas we find it entirely wanting in those which breathe by branchiæ and membranous lungs. In hybernating animals, also, the thymus exhibits alternations of enlargement and decrease, according to the state of the respiratory organs. In the amphibia it attains its maximum of development. The circumstance too of the gland being usually rather larger than ordinary in phthisical patients, may be mentioned as lending some probability to the view proposed.—*Half Yearly Abstract.*

